# PhaGAA: an integrated web server platform for phage genome annotation and analysis

**DOI:** 10.1093/bioinformatics/btad120

**Published:** 2023-03-06

**Authors:** Jiawei Wu, Qingrui Liu, Min Li, Jiliang Xu, Chen Wang, Junyin Zhang, Minfeng Xiao, Yannan Bin, Junfeng Xia

**Affiliations:** Key Laboratory of Intelligent Computing and Signal Processing of Ministry of Education and Information Materials and Intelligent Sensing Laboratory of Anhui Province, and Institutes of Physical Science and Information Technology, Anhui University, Hefei, Anhui 230601, China; Key Laboratory of Intelligent Computing and Signal Processing of Ministry of Education and Information Materials and Intelligent Sensing Laboratory of Anhui Province, and Institutes of Physical Science and Information Technology, Anhui University, Hefei, Anhui 230601, China; BGI-Shenzhen, Shenzhen 518083, China; Shenzhen Key Laboratory of Unknown Pathogen Identification, BGI-Shenzhen, Shenzhen 518083, China; School of Computer Science and Technology, Anhui University, Hefei, Anhui 230601, China; Key Laboratory of Intelligent Computing and Signal Processing of Ministry of Education and Information Materials and Intelligent Sensing Laboratory of Anhui Province, and Institutes of Physical Science and Information Technology, Anhui University, Hefei, Anhui 230601, China; School of Computer Science and Technology, Anhui University, Hefei, Anhui 230601, China; BGI-Shenzhen, Shenzhen 518083, China; Shenzhen Key Laboratory of Unknown Pathogen Identification, BGI-Shenzhen, Shenzhen 518083, China; Key Laboratory of Intelligent Computing and Signal Processing of Ministry of Education and Information Materials and Intelligent Sensing Laboratory of Anhui Province, and Institutes of Physical Science and Information Technology, Anhui University, Hefei, Anhui 230601, China; Key Laboratory of Intelligent Computing and Signal Processing of Ministry of Education and Information Materials and Intelligent Sensing Laboratory of Anhui Province, and Institutes of Physical Science and Information Technology, Anhui University, Hefei, Anhui 230601, China

## Abstract

**Motivation:**

Phage genome annotation plays a key role in the design of phage therapy. To date, there have been various genome annotation tools for phages, but most of these tools focus on mono-functional annotation and have complex operational processes. Accordingly, comprehensive and user-friendly platforms for phage genome annotation are needed.

**Results:**

Here, we propose PhaGAA, an online integrated platform for phage genome annotation and analysis. By incorporating several annotation tools, PhaGAA is constructed to annotate the prophage genome at DNA and protein levels and provide the analytical results. Furthermore, PhaGAA could mine and annotate phage genomes from bacterial genome or metagenome. In summary, PhaGAA will be a useful resource for experimental biologists and help advance the phage synthetic biology in basic and application research.

**Availability and implementation:**

PhaGAA is freely available at http://phage.xialab.info/.

## 1 Introduction

Over the last two decades, antimicrobial resistance has become a global health and development threat. Phages are viruses that kill bacteria specifically but cannot disrupt the normal microflora (Housby and Mann 2009). As a result, phage therapy is a viable replacement for the antimicrobial resistance crisis. As one critical step in phage synthetic biology, phage genome annotation is worthy of attention for the design of phage therapy.

There are various annotation tools that have been developed for phage genomes (Amgarten et al. 2020; Hockenberry and Wilke 2021). But most of these tools just focus on a mono-function, or must be operated in the command-line mode. In recent years, two novel integrated tools have been developed for phage genome annotation. One is a Galaxy and Apollo platform for phage genome annotation and visualization (Ramsey et al. 2020), and the other is Prophage Hunter (Song et al. 2019). The former integrates several bioinformatics tools to analyze phage genomes, but it requires complex procedures with long running time. The latter, Prophage Hunter, mainly identifies active prophages from bacterial genomes and provides simple annotations for these prophages. Though Prophage Hunter is fast and easy to use, the number of integrated tools is very limited. Given all that, to overcome the above-mentioned deficiencies, a user-friendly tool, which integrates more comprehensive functions, is urgently needed for phage genome annotation.

In this work, we developed PhaGAA, an integrated web server platform for phage genome annotation and analysis. Compared with the existing annotation tools, PhaGAA can not only provide more functional annotations (Supplementary Table S1) but also annotate multiple types of input files: phage whole genome, bacterial whole genome, and metagenome assembly.

## 2 Functionalities

The overall framework of PhaGAA is mainly divided into the following four modules (Supplementary Fig. S1 and Supplementary Tutorials):

### 2.1 Input module

In this module, three different types of data could be uploaded and annotated, including phage genome, bacterial genome, and metagenomics assembly. For the phage genomes, the platform annotates these genomes directly. However, for bacterial and metagenomics assembly data, the premise of annotation is to mine potential phage contigs. From these popular tools that provide source code to mine phage contigs, such as PhageBoost (Siren et al. 2021), Phigaro (Starikova et al. 2020), PPR-Meta (Fang et al. 2019), DBSCAN-SWA (Gan et al. 2020), and Seeker (Auslander et al. 2020), we divide these tools according to the mining objectives (for bacterial genome or metagenomics assembly data). For bacterial genome, we selected PhageBoost, which is faster than other similar tools to predict and has been proven to be able to find more prophages. For metagenomics assemblies, we selected Phigaro tool with good performance in terms of operation speed and PPV indicators.

### 2.2 DNA-based module

With the given phage genomes, a DNA-based module is used to annotate the following basic information of the query phages.

1. Assessing quality
  For the prophage sequences mined from metagenome or bacterial genome, CheckV (Nayfach et al. 2021) is used to check their quality in relation to genome completeness and host sequence contamination. They are five quality levels based on the prediction result: complete (100% completeness), high quality (>90% completeness), medium quality (50%–90% completeness), low quality (0%–50% completeness), and undetermined quality (no completeness estimate available).
2. Predicting host
  Phages are highly specific for their host, which is the bacterial targets. By integrating a deep neural model vHULK (Amgarten et al. 2020), this module could provide the information (species and genera) of phage-specific host. Comparing with the other tools for predicting phage hosts [RaFAH (Coutinho et al. 2020), VHM-net (Wang et al. 2020), and CRISPR spacers (Pourcel et al. 2020)], the average precision of vHULK (across all hosts) is four times higher than the second best method CRISPR spacers at species level, and vHULK also outperforms the other tools at generic level. Currently, the predicted results cover 52 species and 61 genera of prokaryotic host.
3. Searching for the closest phage
  The closest phage search is an important approach to suggest the possible functions of unknown phages. FastANI (Jain et al. 2018) is a tool to quickly calculate the whole genome ANI using alignment-free approximate sequence mapping. Compared with BlastN (Ye et al. 2006) method, FastANI achieves much higher accuracy and speedup. FastANI assumes a probabilistic identity cutoff that is set to 80% by default. If ANI value is <80%, there is no output for a genome pair. So, the direct quality comparison is performed between query phages and target phages with ANI value >80%. The target phages are provided from the self-construct phage genome database (15 709 whole phage genomes download from NCBI). When ANI value <80%, the quality comparison should be performed at amino acid level with EzAAI (Kim et al. 2021). EzAAI is a suite of workflows for improved AAI calculation performance. However, due to the limitation of the server configuration, it takes more than 10 h per phage genome with EzAAI. Thus, we do not integrate this tool in PhaGAA platform.
4. Recognizing lifestyle
  Phage lifestyles are classified into temperate and virulent categories. The latter has particularly important implications for phage therapy design. To date, the popular tools for predicting phage lifestyle include PhageAI (Tynecki et al. 2020), BACPHLIP (Hockenberry and Wilke 2021), Deephage (Wu et al. 2021), and PhaTYP (Shang et al. 2022). For PhageAI, there is only web server with a limit of usage times. For BACPHLIP, only complete phage genomes could be predicted. Though DeePhage and PhaTYP can predict the lifestyle of both complete phage genome and phage short contigs, PhaTYP achieves the better performance than DeePhage. Therefore, PhaTYP is integrated into the PhaGAA platform to predict the lifestyle categories of the given phage genomes or contigs.
5. Predicting promoter
  Promoter is a key DNA element in the regulatory network and could modulate the gene expression, which is important on phages infection strategy. At present, there are only two methods for phage promoter prediction, PhagePromoter (Sampaio et al. 2019) and DPProm (Wang et al. 2022). Compared with PhagePromoter, DPProm can effectively reduce false positive rate. As a result, we integrate the two-layer model DPProm, and the promoters and their types on phage genomes could be mined in our platform.
6. Finding candidate spanin genes
  Spanin proteins are essential for lysing the inner and outer membranes of bacteria (Kongari et al. 2018). Thus, we used a tool from the Galaxy and Apollo platform to annotate the potential spanin genes from phage genomes.

### 2.3 Translation module

Translation from phage genomes to the various protein sequences is a critical step in genome annotation, and this step is processed through open reading frames (ORFs) identification. Compared with several tools [Prodigal (Hyatt et al. 2010), Glimmer (Delcher et al. 1999), and GeneMarkS (Besemer et al. 2001)], Prokka (Seemann 2014) obtains high accurate results for identifying open reading frames in prokaryotes. However, compared with prokaryote genomes, phage genes are usually shorter, with extremely compact genomes, overlapping adjacent genes, and genes completely located in other longer genes. As a novel approach for gene identification that is specifically designed for phage genomes, PHANOTATE (McNair et al. 2019) could find more potential ORFs than Prokka. These ORFs may encode novel proteins that have been missed by existing gene callers designed to annotate bacterial genomes. Therefore, we use PHANOTATE to obtain the corresponding coding sequences.

### 2.4 Protein-based module

With the given protein sequences obtained from the translation module, this protein-based module is performed to predict the following functional information of the query phages.

1. Identifying phage virion protein
  Phage virion proteins (PVPs) could perforate the membrane of bacterial hosts and then the replicated phages will release in host cells. Meta-iPVP is a sequence-based meta-predictor employing probabilistic information for the accurate identification of PVPs, and outperforms the other existing PVP predictors (Charoenkwan et al. 2020). Thus, we integrate Meta-iPVP in our platform.
2. Classifying structural proteins
  We use a machine learning-based multiclass classification method PhANNs (Cantu et al. 2020) to classify structural proteins, which is the only tool for predicting structural proteins in phages. PhANNs classify phage proteins into structural proteins (include 10 structural types: major capsid, minor capsid, baseplate, major tall, minor tail, portal, tail fiber, tail sheath, collar, and head–tail joining) or nonstructural proteins.
3. Recognizing protein domain
  As the structural unit, domain is also important to understand the function of the phage protein. We recognize functional domains of proteins by employing the Pfam database (Jones et al. 2014).
4. Classifying functional protein
  The mutation of the same ancestral gene expresses various proteins with similar structures and different functions. As a database specifically designed for prokaryotic viruses, PHROGS (Terzian et al. 2021) is annotated based on the virus databases and is managed manually by experts, making the results more reliable. Therefore, we use the PHROGs database to realize the task of functional classification.

## 3 Discussion

PhaGAA is an integrated platform for phage genome annotation and analysis. Compared with the other annotated tools, PhaGAA has three advantages: (i) it could process three different types of data; (ii) it could provide more functional annotations, such as lifestyle recognition and host prediction; (iii) the web server platform is convenient for biologists. In order to promote the further development of PhaGAA, we will add more annotation and visualization functions, as well as a variety of tools for users to choose and analyze the prediction results. Furthermore, we will implement user accounts that permit users to log in and save and review their own history and other operations in the future.

## Supplementary Material

btad120_Supplementary_Data
