# Supplementary material for: PhaGAA: an integrated web server platform for phage genome annotation and analysis
Source: Bioinformatics. 2023 Mar 6;39(3):btad120. doi: 10.1093/bioinformatics/btad120 (PMC10013646; doi:10.1093/bioinformatics/btad120)
Supplement: btad120_Supplementary_Data [file btad120_supplementary_data.docx]

**PhaGAA: an integrated web server platform for phage genome annotation and analysis**

Jiawei Wu^1^**^†^**, Qingrui Liu^1^**^†^**, Min Li^2^, Jiliang Xu^3^, Chen Wang^1^, Junyin Zhang^3^, Minfeng Xiao^2^*, Yannan Bin^1^* and Junfeng Xia^1^*

^1^Key Laboratory of Intelligent Computing and Signal Processing of Ministry of Education and Information Materials and Intelligent Sensing Laboratory of Anhui Province, and Institutes of Physical Science and Information Technology, Anhui University, Hefei, Anhui 230601, China.

^2^Shenzhen Key Laboratory of Unknown Pathogen Identification, BGI-Shenzhen, Shenzhen, 518083, China.

^3^School of Computer Science and Technology, Anhui University, Hefei, Anhui 230601, China.

**^†^**The authors wish it to be known that, in their opinion, the first two authors should be regarded as Joint First Authors.

*To whom correspondence should be addressed.

Contents

[1. Supplementary Table 2](#_Toc105492736)

[2. Supplementary Figure 3](#_Toc105492737)

[3. Supplementary Tutorials 4](#_Toc105492738)

[3.1 How to use PhaGAA 4](#_Toc105492739)

[3.2 How to understand the annotation report 8](#_Toc105492740)

# Supplementary Table

**Table S1. Comparison of PhaGAA with other annotation tools.**

| Function | PhaGAA | Prophage Hunter | Galaxy and Apollo |
| --- | --- | --- | --- |
| Assessing quality | Yes | No | No |
| Identifying prophage | Yes | Yes | No |
| Recognizing lifestyle | Yes | No | No |
| Predicting host | Yes | No | No |
| Predicting promoter | Yes | No | No |
| Identifying phage virion protein | Yes | No | No |
| Gene calling | Yes | Yes | Yes |
| Classifying structural protein | Yes | No | No |
| Searching for closest phage | Yes | Yes | Yes |
| Classifying functional protein | Yes | Yes | Yes |
| Recognizing protein domain | Yes | Yes | No |
| Finding candidate spanin gene | Yes | No | Yes |

# Supplementary Figure


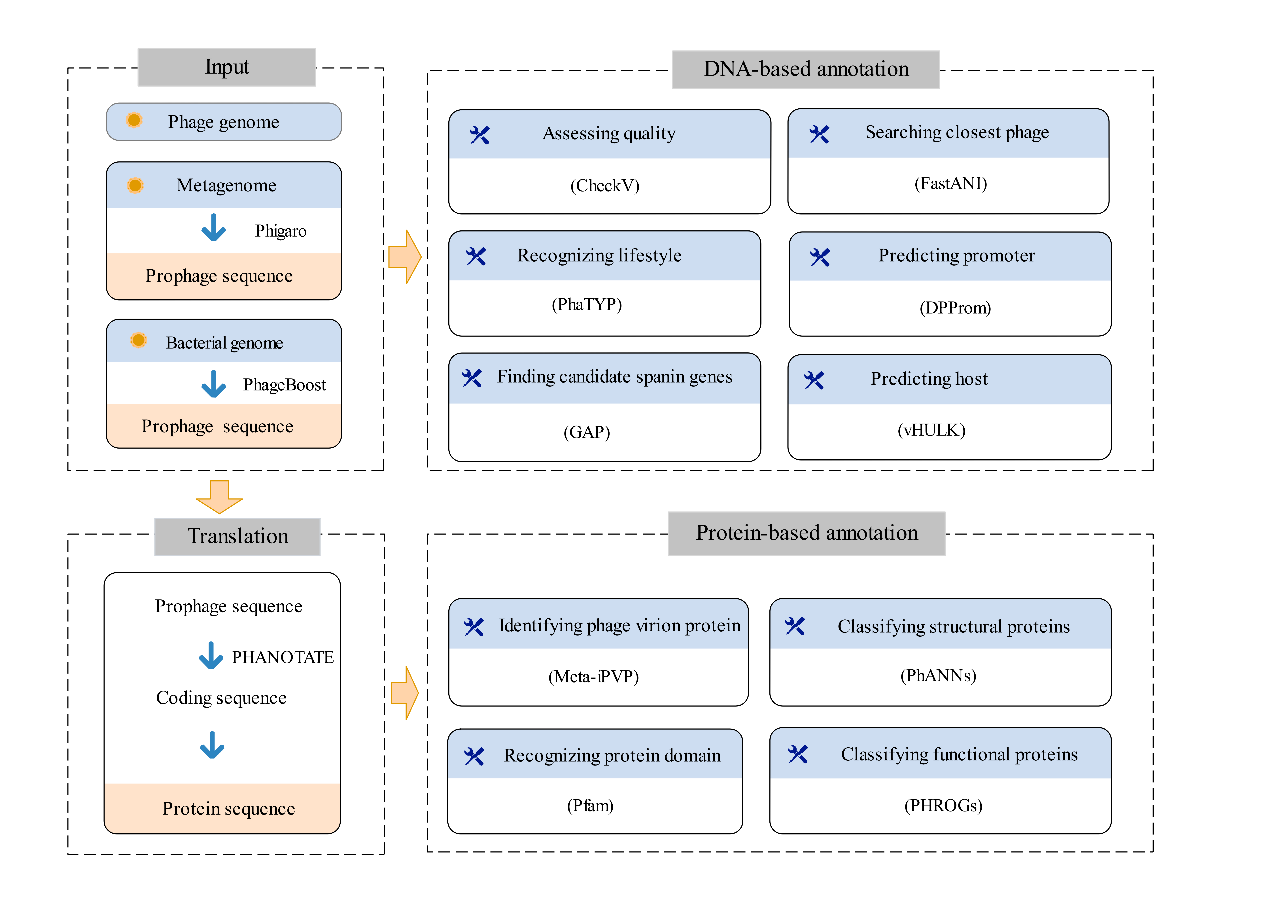


**Figure S1.** The overall framework of PhaGAA.

# Supplementary Tutorials

## How to use PhaGAA

1. Click on the “Predict” option on the navigation bar of the web page to launch the phage annotation platform (Fig .1).


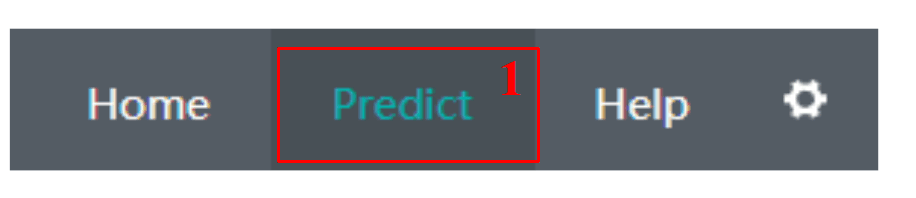


Fig. 1. “Predict” option on the navigation bar.

1. Select the type of uploaded data (Fig. 2).


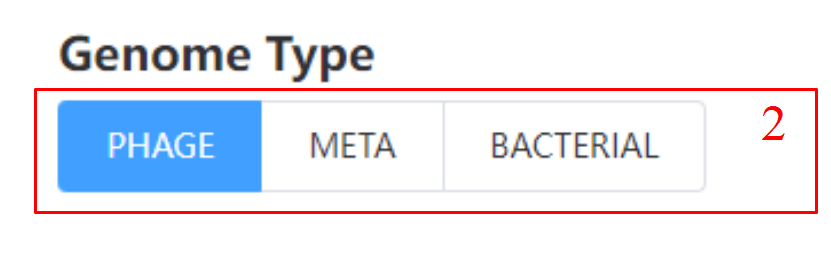


Fig. 2. Three different types of data could be uploaded.

- **PHAGE (Phage Genome):** the phage genome sequence file (FAST format only)
- **META (Metagenome Assembly):** the metagenome assembly sequences file (FAST format only)
- **BACTERIAL (Bacterial Genome):** the bacterial genome sequences file (FAST format only)

1. Select one or more functions to be annotated (Fig. 3.).


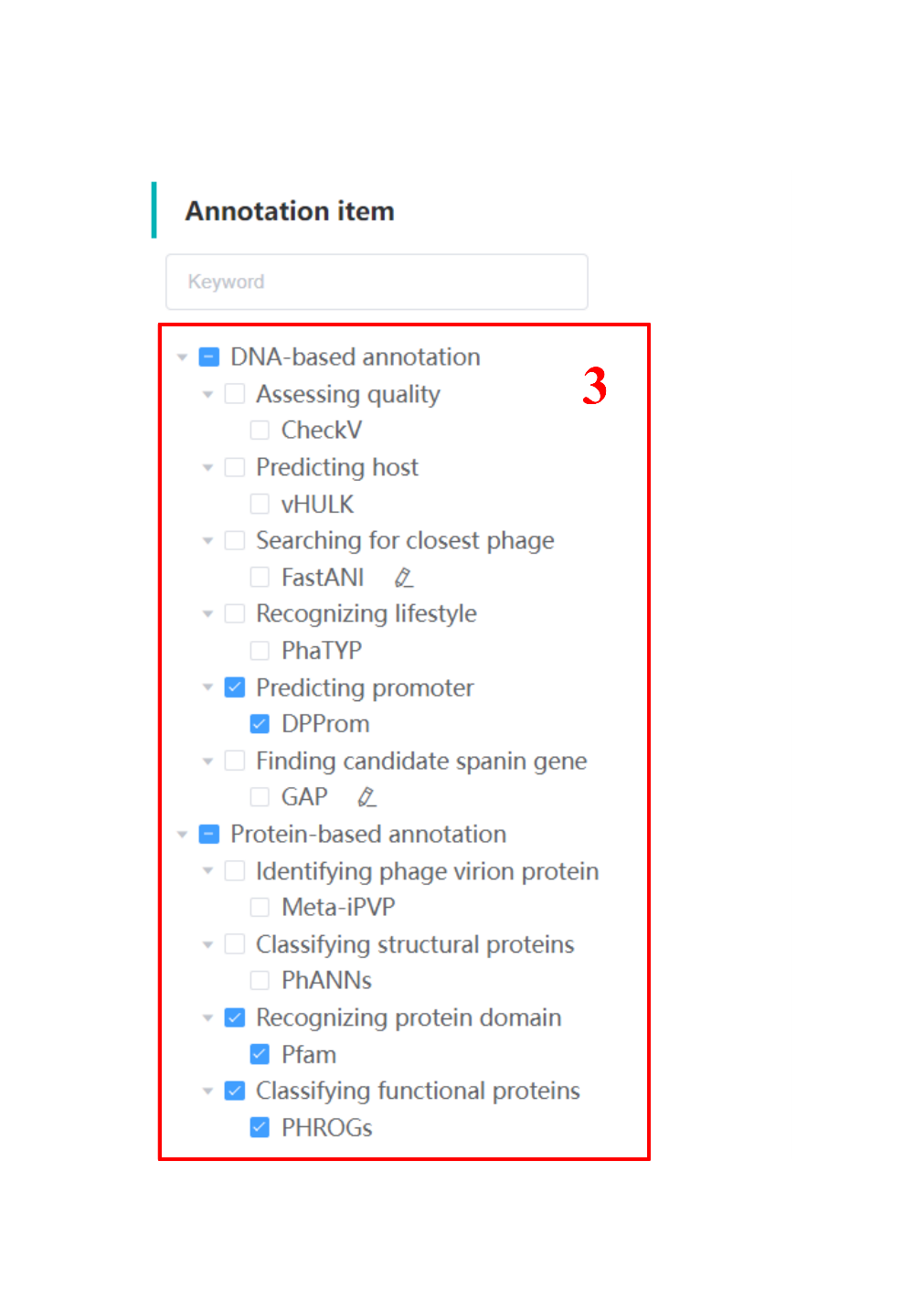


Fig. 3. Different annotated functions in PhaGAA.

At present, only FastANI and GAR tools can allow users to customize their parameters. The parameter setting interface of FastANI and GAP are shown in the following figures.


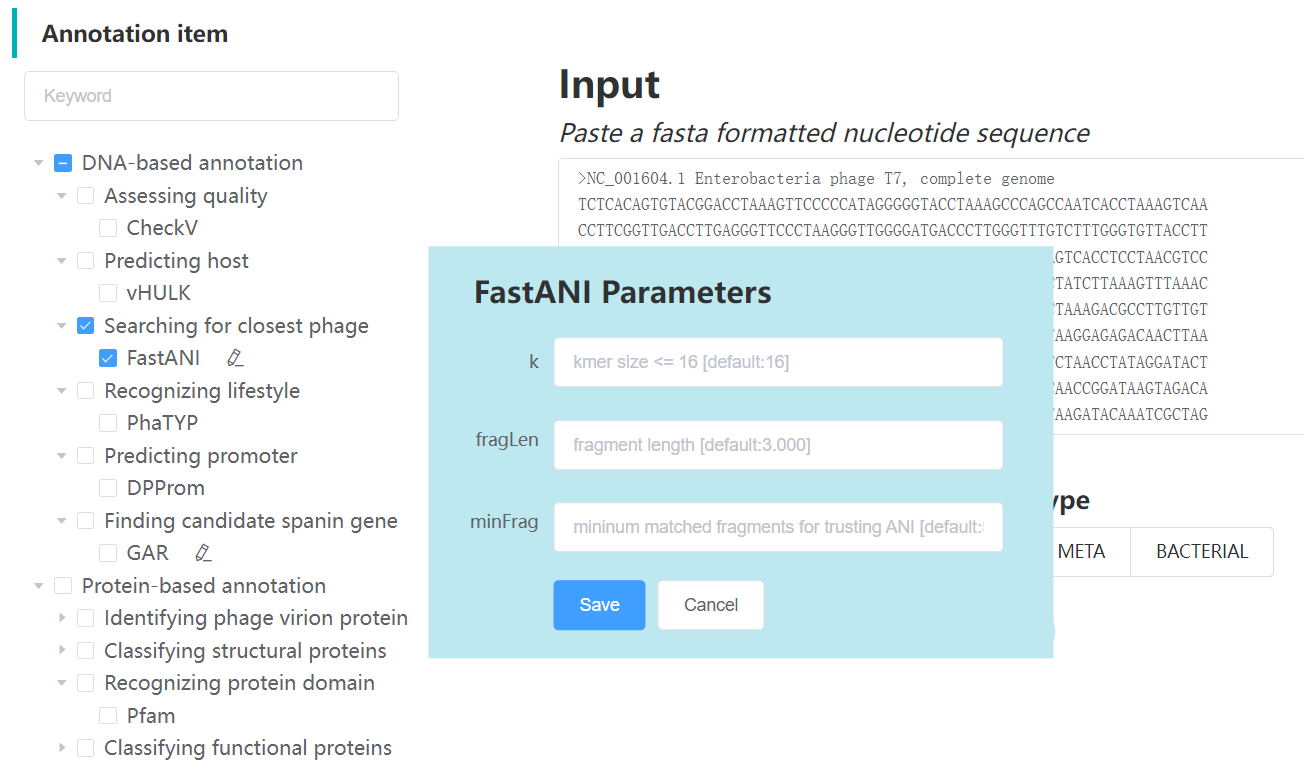


Fig. 3.1 Parameter setting interface of FastANI tool.


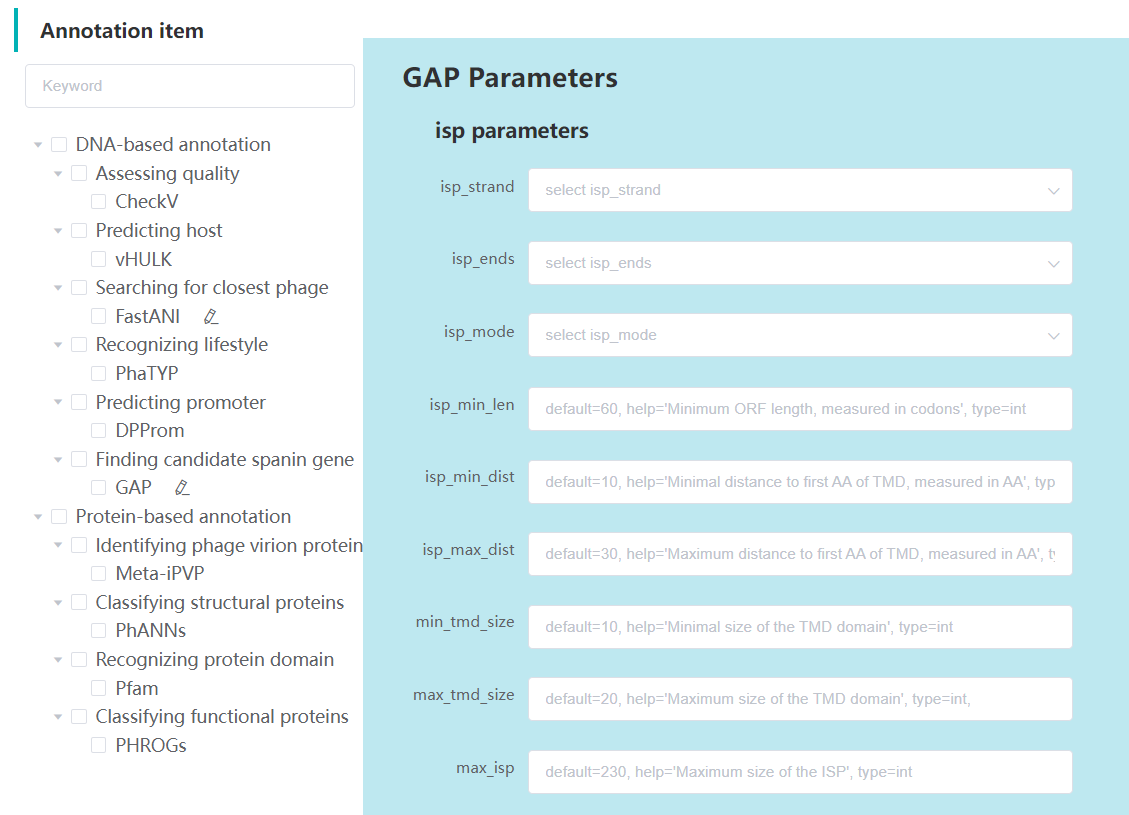


Fig.3.2 Parameter setting interface of GAP tool.

Details of all parameters are as follows:

- FastANI tool:
- k: kmer size <=16.
- fragLen: fragment length.
- minFrag: minimum matched fragments for trusting ANI.
- GAP tool:
- strand: select strand, choices in ("both", "forward", "reverse").
- ends: open or closed. Closed ensures start/stop codons are present, choices in ("open", "closed").
- mode: output all ORFs/CDSs from sequence, all ORFs/CDSs with max length, or first with maximum length, choices in ("all", "top", "one").
- min_len: minimum ORF length, measured in codons.
- min_dist: minimal distance to first AA of TMD, measured in AA.
- max_dist: maximum distance to first AA of TMD, measured in AA.
- Min_tmd_size: minimal size of the TMD domain.
- max_tmd_size: maximum size of the TMD domain.
- max_isp: maximum size of the ISP.
- max_osp: maximum size of the OSP.
- peri_min: amount of residues after TMD is found min.
- peri_max: amount of residues after TMD is found max.
- min_lipo_after: minimal amount of residues after lipobox.
- max_lipo_after: maximum amount of residues after lipobox.

1. Enter your email address to receive the analysis report (Fig. 4**)**.


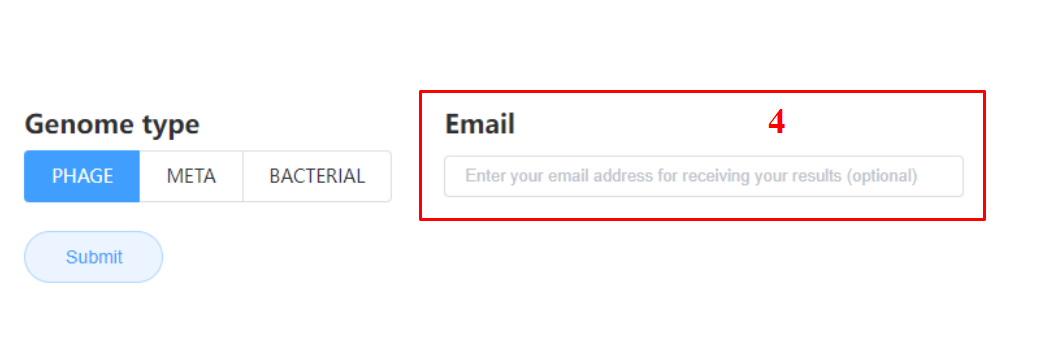


Fig. 4. Email address option.

The four function annotations (“Predicting promoter”, “Classifying structural proteins”, “Recognizing protein domain” and “Protein functional classification”) are time-consuming. If you annotate the four functions, it is recommended to provide your email address to in the receive the analysis report. Once the annotation process finishes, the link of the analysis report will be automatically sent to the email.

Note: the link is valid for one week.

1. Provide data or file to be annotated (Fig. 5).


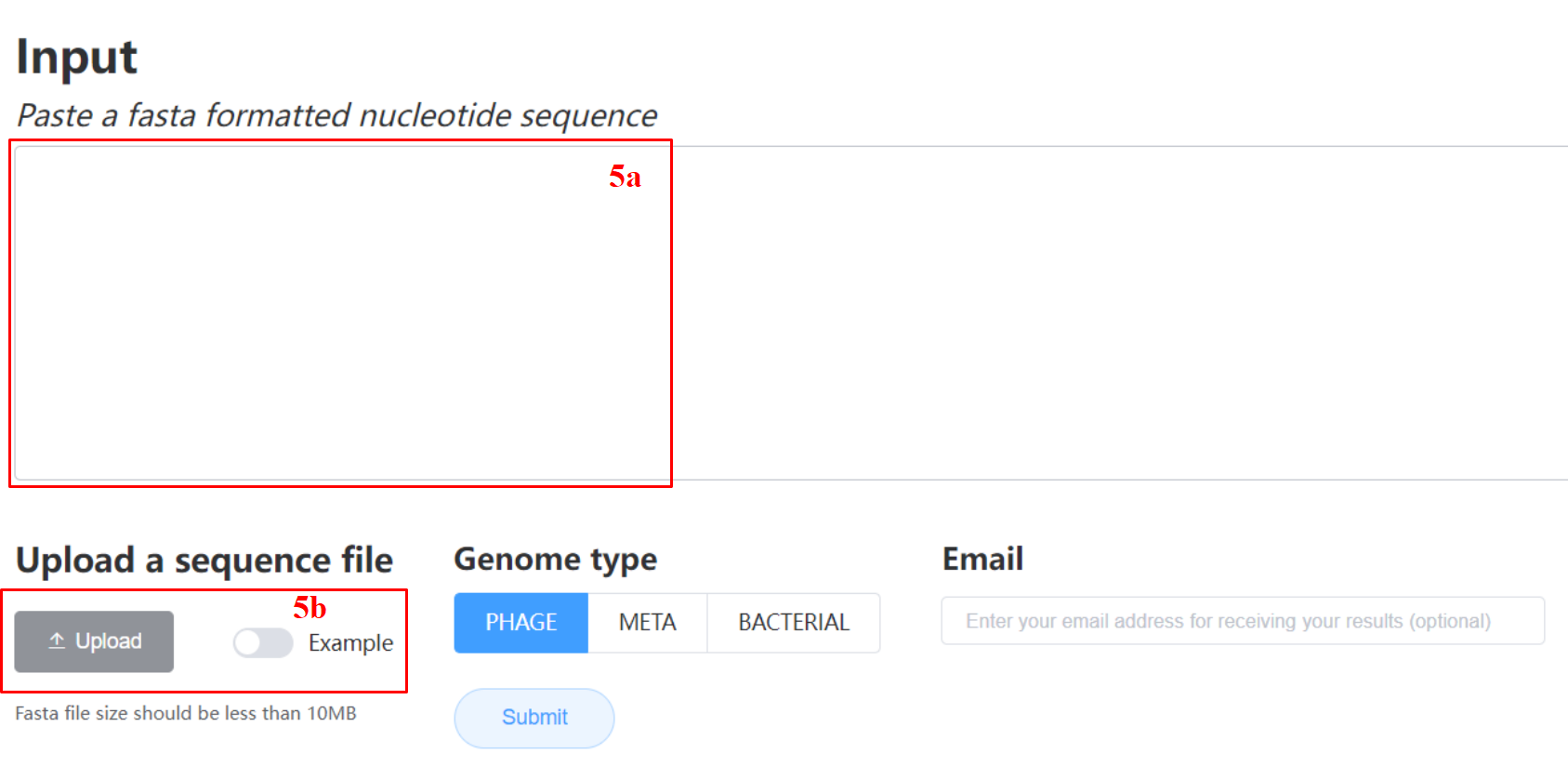


Fig. 5. Different input ways for the genome sequence to be annotated.

Note:

- If you click the “Example” button under the situation of “Genome type: PHAGE”, the annotation of a phage example will be provided in the new page.
- In this platform, phage genome could be annotated with batch processing that’s several phage genomes could be annotated at a time. For the data types of “META” or “BACTERIAL”, only one genome sequence could be processed at a time.
- The size of the uploaded file cannot exceed 10M.

1. Click the “Submit” button to start running (Fig. 6).


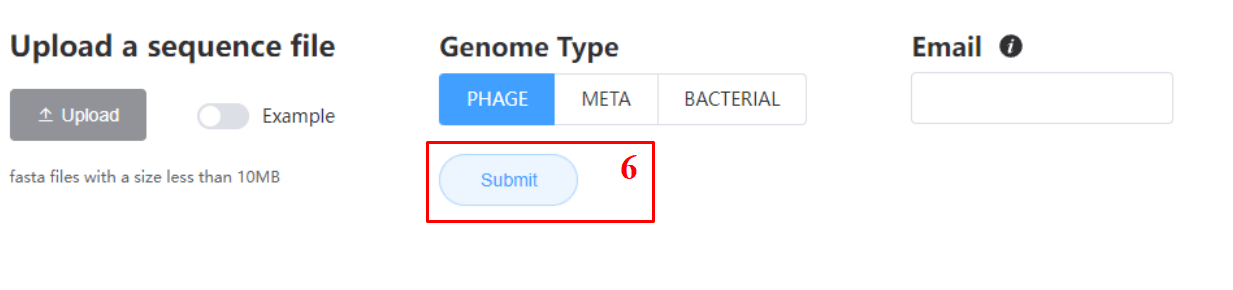


Fig. 6. The submission interface.

1. Wait for the annotation report (Fig. 7).


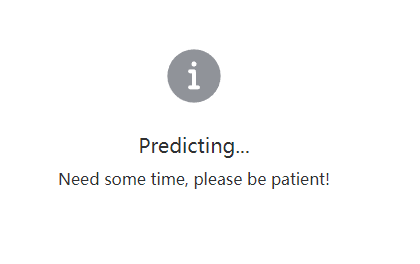


Fig. 7. Wait until the analysis is completed.

If you provide email address, when the annotation is finished, the link to the analysis report will be automatically sent to the email.

## How to understand the annotation report

1. Basic annotation result (Fig. 8):


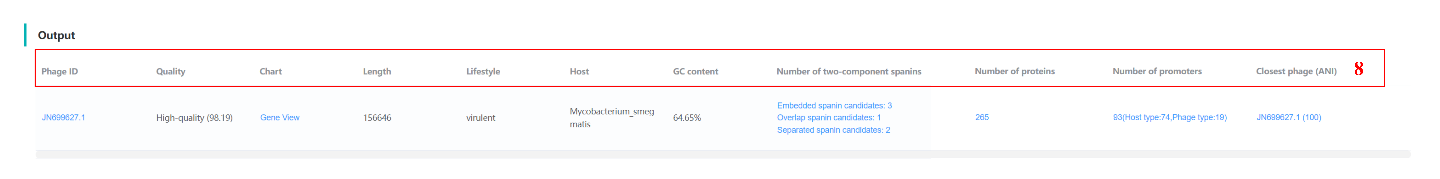


Fig. 8. The annotation results of the phage genomes.

- Phage ID**:** ID of the annotated phage genome;
- Quality: Assessment of phage genome completeness;
- Chart: Visual display of phage genome;
- Length: length of phage genome sequence (unit bp);
- Lifestyle: life cycle of phage (virulent or temperate). Temperate phages lyse the bacterial hosts with a latent phase, whereas virulent phages replicate and lyse the host cell directly and immediately;
- Host: species and genera of host;
- GC content: the ratio of guanine and cytosine to the overall number of bases in the whole-genome sequence;
- Number of two-component spanins: the number of three two-component spanins (Embedding, Overlapping and Separated);
- Isp orf: open reading frame of i-spanin (inner membrane spanin)
- Isp start: starting position of i-spanin gene
- Isp end: ending position of i-spanin gene
- Osp orf: open reading frame of o-spanin (outer membrane spanin)
- Osp start: starting position of o-spanin gene
- Osp end: ending position of o-spanin gene
- Number of proteins: the number of proteins in the whole-genome;
- Number of promoters: potential promoters in phage genomes and their types (“Host type” or “Phage type”);
- Type: promoter type (Host or Phage)
- Start-End: the location of the promoter in the genome sequence
- Length: the length of promoter sequence
- Score: prediction scores of promoter model
- Sequence: promoter sequence
- Closest phage (ANI): the closest phage of the predicted phage compared to the known phages, and “N/A” represents that no target phage with more than 80% similarity to the query phage ANI is found from the self-construct phage genome database;

1. Function annotation results:

Click the number of proteins (Fig. 9), it will display the function information for the proteins (Fig. 10)


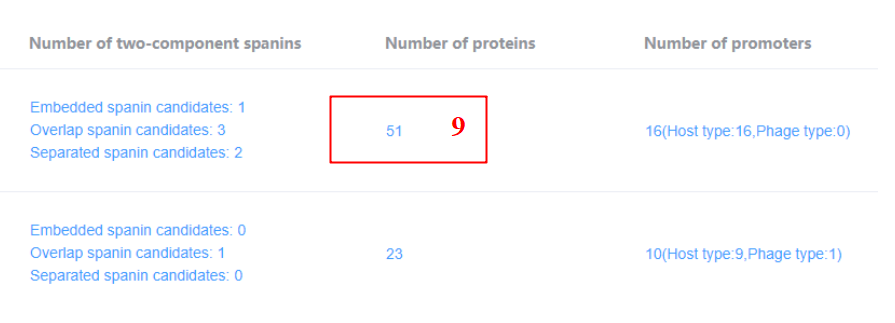


Fig. 9. The number of proteins.


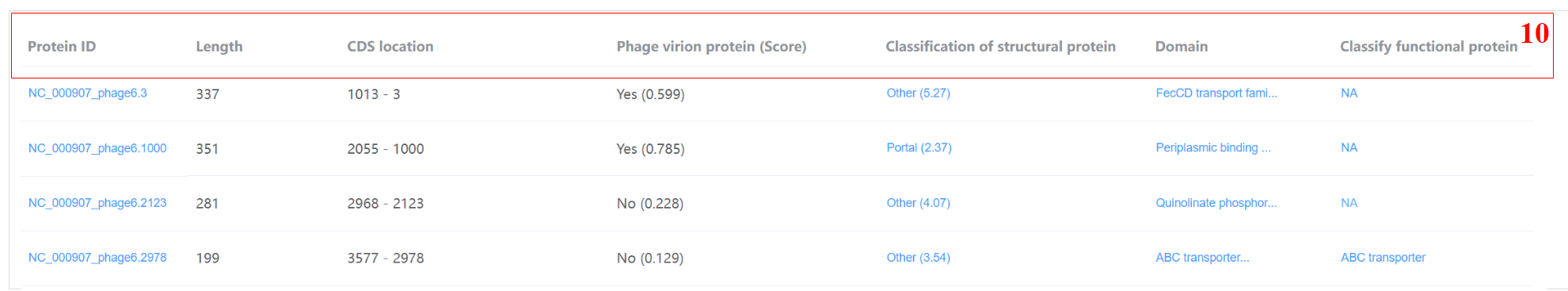


Fig. 10. The details of each annotated protein.

- Protein ID: ID of the protein;
- Length: length of protein sequence;
- CDS location: the position of the gene encoding protein in the phage genome;
- Phage virion protein (Score): whether the protein is phage virion protein. “Score” represents the prediction probability;
- Classification of structural protein: the protein was classified into one of the ten structural proteins (major capsid, minor capsid, baseplate, major tall, minor tail, portal, tail fiber, tail sheath, collar and head-tail joining) or non-structural proteins (other). “Score” represents the prediction probability;
- Domain: the descriptions of protein domain;
- Accession: domain number matched in the Pfam database
- Description: description for the domains of the protein
- Start-End: positional information of the domains
- E-value: reliability evaluation of results. The lower the value, the more reliable the result
- Classify functional protein;
- Phrog id: id of protein orthologous groups in PHROGs database
- Phrog protein: the best matched protein in protein orthologous groups
- Prob: score of the best match
- Annotation: functional description of protein in PHROGs database

1. Phage genome visualization


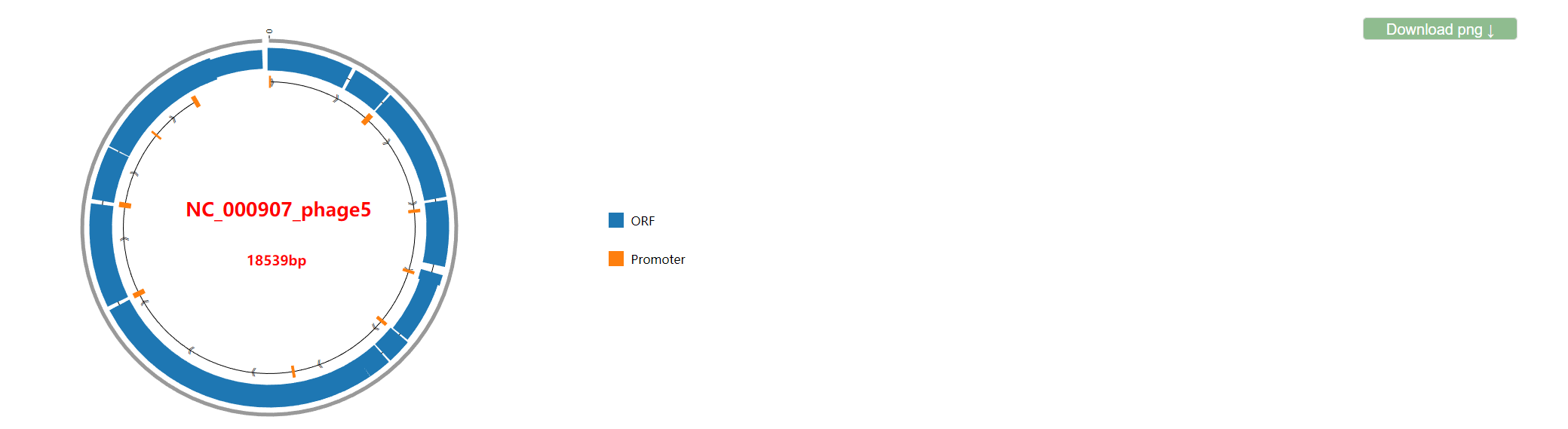


Fig. 11. Visualization picture of phage genome.
